# Early vs. late definitive fixation of pelvic ring fractures in resuscitated polytraumatized patients: a systematic review and meta-analysis

**DOI:** 10.1007/s00402-025-06152-9

**Published:** 2025-12-26

**Authors:** Krishna Oochit, Mohammed Araiz Imran, Andrew Marsh

**Affiliations:** 1 Medical School, University College London, London, UK; 2 Royal Free Hospital, London, UK; 3 Royal Alexandra Hospital, Paisley, UK; 4 Queen Elizabeth University Hospital, Glasgow, UK

**Keywords:** Pelvic fractures, Fracture fixation, Major trauma, Timing of surgery

## Abstract

**Background:**

The aim of this systematic review is to compare the short-term clinical outcomes between early (EDF) and late definitive fixation (LDF) in polytraumatized patients with pelvic ring fractures (PRF).

**Method:**

In accordance with PRISMA guidelines, a comprehensive search using Boolean operators was performed in June 2022 from the following databases: Embase, Medline and Cochrane Library. Studies comparing EDF and LDF for PRF in polytrauma patients defined as ISS > 15 were included. All included cohorts performed definitive fixation after haemodynamic stabilisation or adequate response to resuscitation. Random effects meta-analyses of pooled raw data were employed using the Mantel-Haenszel and Inverse -variance methods. The methodological quality of studies was assessed using the Newcastle Ottawa Scale.

**Results:**

Out of 869 studies screened, 10 were included in the meta-analysis with a total of 2918 patients. All included studies were retrospective; no randomized trials were identified. The most common time point used by 7 studies to define EDF was within 24 h of hospital admission and LDF (> 24 h). The most common reasons for LDF were surgeon’s choice, availability of pelvic surgeon and transfer from other hospitals. Our meta-analysis revealed that EDF was associated with a reduced length of hospital stay (WMD=-3.52 days; 95% CI: [-5.43 to -1.62], *p* < 0.0003) and lower incidence of ARDS (RR = 0.50; 95% CI: [0.26 to 0.96], *p* = 0.04). No significant association was found in mortality, length of ICU stay, multi-organ failure, sepsis and surgical site infection between EDF and LDF.

**Conclusion:**

These findings suggest that early definitive fixation may be a safe and viable option with no increased risk of complications and mortality. However, the adequacy of resuscitation and the estimate of physiologic reserve should be balanced with the risks of operative fixation in all patients. Further prospective validation studies are warranted to test the predictive ability of the various proposed trauma care models and stratify patients for EDF.

**Supplementary Information:**

The online version contains supplementary material available at 10.1007/s00402-025-06152-9.

## Introduction

Pelvic fractures are often a result of high-energy trauma and account for around 3 to 8% of all traumatic fractures [1]. They are associated with a significant morbidity and mortality [2]. Most patients are usually polytraumatized and haemodynamically unstable at presentation posing a major challenge to their management [3, 4].

The timing of definitive fixation of pelvic fractures in polytrauma patients is a controversial topic in the trauma literature [5, 6]. To date, most of our knowledge is extrapolated from studies related to the timing of femoral shaft fracture fixation in polytrauma patients [7–9]. Recommendations for early definitive fixation as opposed to a damage control approach with delayed definitive fixation are currently based on the haemodynamic status and response of these critically ill patients to resuscitation, with the advantages of early fracture fixation weighed against the risks of excessive surgical burden [10–13]. Other factors to be considered include the fracture pattern, presence of other injuries and the immunoinflammatory status of patients [14].

The damage control approach is a universally accepted strategy for unstable and in extremis patients as it spares them from the potentially lethal systemic inflammatory cascade known as the ‘second hit’ phenomenon [12, 15, 16]. The concept of early definitive fixation in borderline and stable patients is however still debated and there remains an overzealous application of the damage control approach to these categories of patients [6, 8, 9, 12]. A randomised trial by Bone et al. has failed to demonstrate a benefit of damage control orthopaedics in borderline patients with femur fractures [7]. Several other large studies have shown a reduction in the length of intensive care stay, hospital stay, and complication rates in patients having early definitive fixation after adequate resuscitation [8, 17–20].

The aim of this systematic review is to assess the impact of timing to definitive fixation on short-term clinical outcomes in polytraumatized patients with pelvic fractures by synthesising the available evidence and aims to guide future research on this topic.

## Methods

### Literature and search strategy

This review was performed in accordance with the PRISMA guidelines [21]. A comprehensive search of the following databases: Medline (1946–2024), Embase (1947–2024) and Cochrane Controlled Register of Trials (CENTRAL) was carried out in Dec 2024 using a combination of Medical Subject Headings terms, free text and Boolean logical operators. We used the EXPLODE function to capture narrower terms. No language or time restrictions were applied. The detailed search strategy is provided in Supplementary appendix. The bibliographies of included studies were also manually searched to identify other relevant studies.

### Study selection criteria

All studies identified were extracted into the Mendeley referencing manager software©. A two-stage strategy was used to sift through all the articles:

Stage 1: Two independent authors screened through the titles and abstract to identify relevant studies.

Stage 2: All studies identified from stage 1 underwent full text screening by two independent authors and were included if they satisfied our inclusion/exclusion criteria. Where discrepancies arose at any of those stages, they were resolved by consensus after the full text of the article was reviewed and discussed with a third author.

Inclusion criteria:

1. Clinical studies containing original data (randomised controlled trials, prospective cohort studies and retrospective studies).
2. Studies having 2 timing cohorts (early definitive fixation vs. delayed definitive fixation) reporting mortality, complications, length of hospital or ICU stay)
3. High energy pelvic fractures in polytrauma patients (Age > 16).

Exclusion criteria:

1. Case reports, conference abstracts, reviews, guidelines, comments and studies involving less than 10 patients.

### Data extraction and quality assessment

Data was extracted into a Microsoft Excel sheet. The main data points included: study details [author, publication date, study design, country], number of patients, baseline data [age, Injury Severity Score (ISS), Abbreviated injury Severity Scores (AIS), Glasgow Coma Scale (GCS)] and outcome data in both the early definitive fixation (EDF) and late definitive fixation (LDF) groups [mortality, overall hospital and intensive care unit (ICU) stay, Acute Respiratory Distress Syndrome (ARDS), deep vein thrombosis (DVT), pulmonary embolism (PE), multiorgan failure (MOF), sepsis, surgical site infections and any other complications]. *Mortality was* described when a patient succumbed during their first hospital stay after surgery.

The methodological quality of the included studies was independently assessed using the Newcastle Ottawa Scale (NOS) by two reviewers [22]. The NOS uses three domains: study selection, comparability, and outcome/exposure to assess the quality of non-randomized studies. The scores obtained were then classified as good (7–9 stars), fair (4–6 stars) and poor quality (0–3 stars) as per the Agency for Healthcare Research and Quality standards. We performed an interrater reliability analysis using Cohen’s Kappa to determine consistency between the two reviewers.

### Statistical analysis

The Review Manager Software 5.4 and SPSS Statistics Windows 28.0 (IBM, Armonk, NY) were used to perform statistical analyses. Random effects meta-analyses of pooled raw data were employed using the Mantel- Haenszel and Inverse-variance statistical method for each outcome with sufficient data to reduce heterogeneity, in line with the Cochrane review methodology [23]. Where median and IQR were reported, the mean and standard deviation (SD) were calculated using Wan et al.’s adaptive method [24]. Where standard errors were reported, these were converted to SD by multiplying them by the square root of the sample size. Dichotomous outcomes are presented as risk ratio (RR) with 95% confidence interval (CI) and weighted mean difference (WMD) with 95% CI was calculated for continuous outcomes. The results are presented in forest plots.

Heterogeneity was quantitatively evaluated by the Chi-square test to obtain the I² estimates representing the variability across studies. These are classified as low (< 50%), moderate (50–70%) or high heterogeneity (> 70%). A P value < 0.05 was considered as significant.

## Results

### Search results and characteristics of included studies

**Fig. 1:**
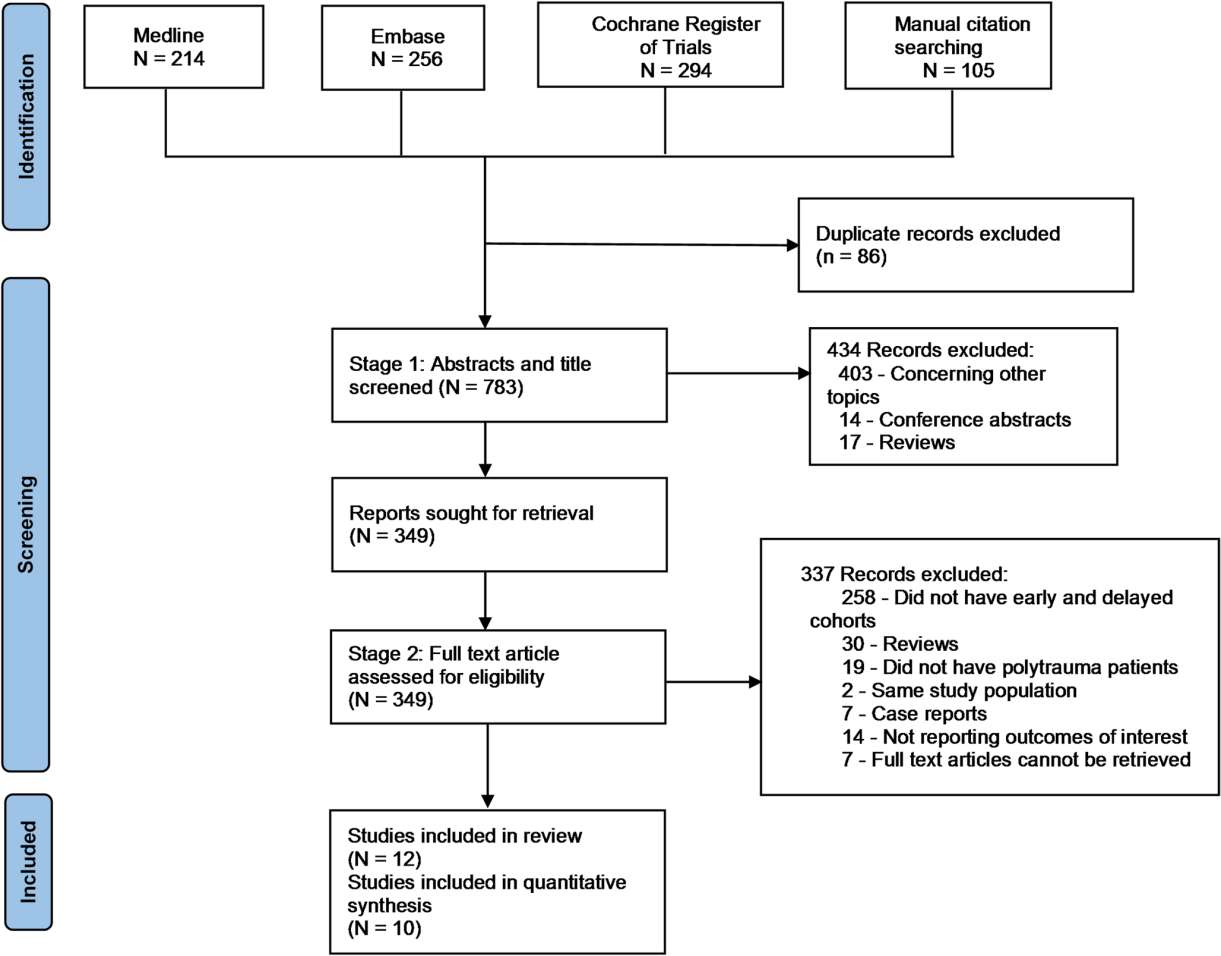
Prisma flowchart of included studies. From: Page et al. [21]

A total of 869 studies were identified from databases, registers and manual citation searching. Out of those 12 studies fulfilled our inclusion criteria [18, 25–35]. Figure 1 depicts our two-stage retrieval strategy and the reasons for exclusion at each stage. The characteristics of the included studies are summarised in Table 1. Eleven were retrospective studies of prospectively collated databases and one was a prospective cohort study. Three studies came from the same centre but were from different study periods with little overlap between them [28, 30, 32]. None of the studies were randomised. They included a total of 2918 patients with 1554 pelvic ring fractures. Table 2 is a narrative synthesis of the included studies.

**Table 1:** Characteristics of included studies

Studies	Year	Country	Study design	Total number of patients	Number of patients in EDF cohort	Number of patients in LDF cohort	Study quality using NOS	Oxford level of evidence
Goldstein	1986	USA	Retrospective	33 patients; 33 PRF	15	18	Poor	3c
Scalea	1999	USA	Retrospective	171 patients; Mixed (majority PRF)	147	24	Fair	3b
Plaisier	2000	USA	Retrospective	100 patients 41 PRF & 59 AF Only PRF analysed	16	25	Fair	3c
Connor	2003	USA	Retrospective	99 patients; 99 PRF	71	28	Good	3b
Vallier	2010	USA	Retrospective	645 patients; 291 PRF 399 AF	165	253	Good	3b
Enninghorst	2010	Australia	Retrospective	45 patients; 45 PRF	18	27	Good	3b
Vallier	2013	USA	Retrospective	1005 patients; 259 PRF 266 AF	111 (572)	148 (433)	Good	3b
Han	2014	China	Retrospective	72 patients; 72 PRF	33	39	Good	3b
Vallier	2015	USA	Prospective cohort study	335 patients; 71 PRF 57 AF	269	66	Good	3b
Acker	2016	Israel	Retrospective	108 patients; 108 PRF	50	58	Good	3b
Rojas	2021	USA	Retrospective	118 patients; 118 PRF	36	82	Good	3b
Taylor	2022	USA	Retrospective	287 patients; 287 PRF	179	108	Fair	3b

PRF: pelvic ring fractures, AF: acetabular fractures, EDF: early definitive fixation, LDF: late definitive fixation, NOS: Newcastle Ottawa Scale

**Table 2:** Narrative synthesis of included studies

Reference	Fractures Included	Time Points			Outcomes		
			Early	Late		Early	Late
Goldstein 1986 [25] Retrospective Timing is from admission	All PRF treated with ORIF	*N* ISS, mean Pelvic angiography (*n*, %) Embolized Laparotomy before ORIF	≤ 72 h 15 41 (14–68) 8 (53%) 5 (33%) 2 (13%)	> 72 h 18 27 (11–50) 6 (33%) 3 (17%) 1 (5.6%)	H LOS, d (mean; range) Vent, d Mortality, % Surgical site infection, % DVT, % Pulmonary complications, %	32 (10–90) 3 (0–14) 1 (6.7%) 2 (13%) 2 (13%) 2 (13%)	37 (10–95) 1 (0–2) 0 5 (28%) 0 4 (22%)
Scalea 1999 [27] Retrospective Timing is from admission	Mixed fractures (Pelvic, femur, tibia): all had head injury	N ISS, (mean; range) A-GCS, (mean; range) AIS head ≥ 3 (n, %) RTS, (mean; range) Required Inotropes (1st 24 h) Required Vasopressors (1st 24 h) Thoracotomy (n, %) Laparotomy (n, %) Craniotomy (n, %) D-GCS, (mean; range)	**≤ 24 h** 147 38 (17–75) 9 (3–15) 122 (76%) 6.2 (2.1–7.8) 35 (24%) 42 (29%) 4 (3%) 32 (22%) 12 (8%) 14 (5–15)	**> 24 h** 24 37.6 (17–68) 10 (3–15) 22 (92%) 6.4 (2.9–7.4) 5 (21%) 7 (29%) 1 (4%) 6 (25%) 1 (4%) 14.5 (11–15)	H LOS, d [Median with IQR] ICU LOS, d Vent, d Required Vent, % Mortality, % CNS complications, %	23 (12–36) 17 (9–29) 12 (5-20.5) 108 (73%) 15 (10%) 6(4%)	22 (12.5–31) 13 (8–24) 11 (5–14) 19 (79%) 4 (17%) 2 (8%)
Plaisier 2000 [27] Retrospective Timing is from admission	PRF and AF; (only PRF data analysed)	N ISS, [mean, (sd)] AIS head [median, (range), n] AIS chest AIS abdomen AIS leg	**≤ 24 h** 16 27.4 (15.5) 2.3, (2–4), 7 3.0, (1–4), 5 2.9, (2–4), 8 3.0, (2–5), 16	**> 24 h** 25 20.4 (9.6) 2.2, (1–3), 6 2.0, (1–4), 9 2.8, (1–4), 12 2.5, (2–4), 25	H LOS, d [Median with IQR] ICU LOS, d	9.0 (7.0-13.5)* 3.5 (1.0-5.5)	14.0 (10.3–23.5) * 2.0 (0.5–6.5)
Connor 2003 [18] Retrospective	All PRF	N ISS, [mean, (sd)]	**≤ 1 week** 71 18.8 (10.2)	**> 1 week** 28 23.2 (13.2)	ICU LOS, d [mean, (sd)] Vent, d Pulmonary complication, %	6.1 (5.1)** 5.4 (6.5)* 9 (12.7%)*^¶^	13.3 (9.0)** 13.5 (9.0)* 8 (28.6%)*
Vallier 2010 [19] Retrospective Timing is from time of injury to surgery	291 PRF and 399 AF (*N* = 645 patients) (418 patients with ISS > 18 included in analysis)	N (ISS > 18) ISS, [mean, (range)] (*N* = 645)	**≤ 24 h** 165 26.9 (9–66)	**> 24 h** 253 24.9 (9–66)	For *N* = 645 H LOS, d [mean, (sd)] ICU LOS, d *For ISS > 18* Mortality, % ARDS, % Pneumonia, % MOF, % All complications, %	10.7 (11.9) 8.1 (7.9) 1 (0.6%) 8 (4.8%)* 14 (8.5%)* 3 (1.8%) 25 (15%)*	11.6 (8.0) 9.9 (9.1) 4 (2.0%) 32 (13)* 43 (17%)* 11 (4.3%) 66 (26%)*
Enninghorst 2010 [29] Retrospective Timing is from admission	All PRF with ISS > 17	N ISS, [mean, (sd)] AIS pelvis, [mean, (sd)] Laparotomy (n, %) Pelvic angiography (n, %)	**≤ 24 h** 18 30 (18) 3.7 (1) 2 (12%) 3 (18%)	**> 24 h** 27 24 (18) 3.4 (1) 2 (8%) 6 (21%)	H LOS, d [mean, (sd)] ICU LOS, d PRBC transfused in 1st 24 h, units Mortality, % Pneumonia, % Surgical site infection, % DVT, %	25 (24) 2.9 (2.5) 4.7 (5) 0 0 0 1 (6%)	37 (32) 3.7 (3.6) 6.6 (4) 3 (11%) 4 (15%) 4 (15%) 2 (8%)
Vallier 2013 [20] Retrospective Timing is from time of injury to surgery	Mixed fractures (259 PRF, 266 AF and femur and spine) All with ISS > 18	N (PRF/total) ISS, [mean, (sd)] AIS chest ≤ 2 (n, %) AIS abdomen ≤ 2 (n, %) AIS chest > 2 (n, %) AIS abdomen > 2 (n, %) Head injury (GCS < 8) (n, %)	**≤ 24 h** 111/572 33.4 (11.4) 99 (17.3%) 87 (15.2%)* 71 (12.4%)* 127 (22.2%)* 75 (13.1%)*	**> 24 h** 148/433 32.5 (11.3) 80 (18.5%) 94 (21.7%)* 141 (32.6%)* 76 (17.6%)* 80 (18.5%)*	H LOS, d [mean, (sd)] ICU LOS, d Vent, d PRBC transfused in 1st 24 h, units Mortality, % ARDS, % Pneumonia, % MOF, % Sepsis, % Surgical site infection, % PE, % DVT, %	10.5 (9.8)* 5.1 (8.8)* 3.0 (7.4)* 5.0 (8.4)* 8 (1.4%) 10 (1.7%)* 49 (8.6%)* 2 (0.35%) 10 (1.7%)* 6 (1.0%) 10 (1.7%) 40 (7.0%)	14.3 (11.4)* 8.4 (11.1)* 5.1 (8.8)* 7.2 (9.1)* 7 (1.6%) 23 (5.3%)* 66 (15.2%)* 1 (0.23%) 23 (5.3%)* 4 (0.92%) 6 (1.4%) 36 (8.3%)
Han 2014 [31] Retrospective Timing is from time of injury to surgery	All PRF (Tile B&C) All Borderline with NISS > 16	N ISS, [mean, (sd)] NISS, [mean, (sd)] GCS, [mean, (sd)] AIS head, [mean, (sd)] AIS chest, [mean, (sd)] AIS abdomen, [mean, (sd)] AIS extremities, [mean, (sd)]	**≤ 24 h** 33 28 (4.5) 30.7 (6.4) 13.6 (1.4)* 0.4 (0.8)* 1.4 (1.5) 2.1 (1.5) 3.6 90.6)	**> 24 h** 39 29.7 (6.0) 33.4 (7.5) 12.6 (1.6)* 0.9 (1.0)* 1.4 (1.6) 2.1 (1.6) 3.6 (0.6)	ICU LOS, d [mean, (sd)] Vent, d ARDS, % Pneumonia, % MOF, % Sepsis, % Surgical site infection, %	2.7 (2.5) 1.6 (0.9) 6 (18.2)* 4 (12%) 7 (21%) 3 (9.1%) 0	3.0 (3.7) 1.7 (1.0) 3 (7.7)* 4 (10%) 5 (13%) 2 (5.1%) 5 (4.5%)
Vallier 2015 [32] Prospective Timing is from time of injury to surgery	Mixed fractures (71 PRF, 57 AF, 173 FF, 79 SF)	N ISS, [mean, (range)] AIS chest ≤ 2 (n, %) AIS abdomen ≤ 2 (n, %) AIS chest > 2 (n, %) AIS abdomen > 2 (n, %) Head injury: GCS 9–15 (n, %) GCS ≤ 8 (n, %)	**≤ 36 h** 269 25.1 (16–66)* 75 (28%) 37 (14%) 92 (34%)* 29 (11%)* 109 (41%) 40 (15%)*	**> 36 h** 66 34 (16–66)* 13 (20%) 12 (18%) 29 (44%)* 19 (29%)* 26 (39%) 17 (26%)*	H LOS, d [mean, (sd)] ICU LOS, d Vent, d Mortality, % ARDS, % Pneumonia, % MOF, % Sepsis, % Surgical site infection, % PE, % DVT, %	9.5 (7.8)* 4.4 (7.0)* 2.6 (5.7)* 4 (1.5%) 4 (1.5%) 22 (8.2%) 1 (0.37%) 6 (2.2%)* 6 (2.2%)* 7 (2.6%) 6 (2.2%)	17.3 (9.1)* 11.6 (8.6)* 7.6 (7.0)* 1 (1.5%) 1 (1.5%) 9 (14%) 1 (1.5%) 12 (18%)* 3 (4.5%)* 1 (1.5%) 0
Acker 2016 [33] Retrospective Timing is from admission	All PRF Tile B&C	N ISS, [mean, (sd)]	**≤ 24 h** 50 23.6 (13)	**> 24 h** 58 20.4 (13)	H LOS, d [mean, (sd)] ICU LOS, d All complications,%	30.3 (27.2) 13 (21.0)* 5 (10%)	21.4 (19.0) 9.6 (10.8)* 3 (5.2%)
Rojas 2021 [34] Retrospective Timing is from time of injury to surgery	All PRF	N ISS, [mean, (sd)] GCS, [mean, (sd)] Head injury (n, %) Chest injury (n, %) Spine injury (n, %) Abdomen injury (n, %) Urologic injury (n, %)	**≤ 36 h** 36 24 (9)* 14 (3) 12 (33%) 12 (33%)* 12 (33%) 6 (17%) 6 (17%)	**> 36 h** 82 28 (9)* 13 (5) 26 (32%) 51 (62%)* 43 (52%) 11 (13%) 20 (24%)	H LOS, d [mean, (sd)] ICU LOS, d Vent, d Mortality, % ARDS, % Pneumonia, % MOF, % Sepsis, % PE, % DVT, %	13 (13) 3 (5)* 1 (3) 1 (3%) 4 (11%) 0 0 2 (6%) 2 (6%) 2 (6%)	17 (14) 7 (9)* 4 (9) 3 (4%) 19 (23%) 16 (20%)* 4 (5%) 4 (5%) 2(2%) 2 (2%)
Taylor 2022 [35] Retrospective Timing is from admission	All PRF	N ISS, [mean, (SEM)] Pelvic embolization (n, %) Lifesaving surgery prior to pelvic fixation (n, %)	**≤ 72 h** 179 14.9 (0.8) 36 (20.1%) 54 (30.2%)	**> 72 h** 108 17.7 (1.1) 18 (16.7%) 29 (26.9%)	H LOS, d [mean, (SEM)] Mortality, % ARDS, % Surgical site infection, % PE, % DVT, %	11.9 (0.7)* 1 (0.6%) 0 5 (2.8%)* 5 (2.8%) 3 (1.7%)	18.0 (1.2)* 0 1 (0.9%) 9 (8.3%)* 1 (0.9%) 6 (5.6%)

### Methodological quality

The overall Kappa score was 0.85 (*p* < 0.001) indicating a good level of agreement between reviewers. The quality of the 12 included studies ranged from 3 to 7 with a mean of 6. There were eight ‘Good’ studies [18, 28–34], three ‘fair’ studies [26, 27, 35] and one ‘poor’ study [25]. (Supplementary Table S1)

### Narrative synthesis

#### Variations in definition of timing of surgery

Among the 12 studies, there were four different timepoints used to define EDF or LDF. The commonest time point for EDF was within 24 h used by seven studies [26–31, 33], two studies [32, 34] used within 36 h, two studies [25, 35] used within 72 h and one study [18] used within 1 week. Six studies [25–27, 29, 33, 35] used timing from hospital admission to definitive surgery as compared to five studies [28, 30–32, 34] using timing of injury to definitive fixation and one study [18] did not specify.

#### Comparability of cohorts between studies

- **ISS status**

Nine studies [18, 26–31, 33, 35] had similar mean injury severity scores (ISS) between the early and late cohorts. One study [25] had higher ISS in the EDF group, and two studies [32, 34] had higher ISS in the LDF group.

- **Reason for choosing late definitive surgery**

Decision for the timing of fracture fixation was based on the judgement of the surgical team in most studies. Among studies reporting specific reasons for choosing late definitive surgeries, the most common ones were the surgeon’s choice [25, 26, 32], availability of pelvic surgeons [25, 29], operation theatres availability [28, 32] and transfers from other hospitals [25, 28, 32].

### Results from meta-analysis

Ten studies with comparable cohorts were pooled into a meta-analysis [25–31, 33–35]. Table 3 is a summary of the meta-analysis.

**Table 3:** Results of meta-analysis using random effects model

Outcomes	Number of studies	Number of patients: EDF	Number of patient: LDF	RR (95% CI)	WMD (95% CI)	*p*-value	Heterogeneity
Mortality	6	1117	927	0.68 [0.36,1.26]	-	0.22	0%
Hospital length of stay (days)				-			
ICU length of stay (days)	7	982	848	-	-0.54 [-2.11, 1.04]	0.50	74%
Acute respiratory distress syndrome	5	985	915	0.50 [0.26, 0.96]	-	**0.04**	45%
Pneumonia	4	252	401	0.48 [0.20, 1.18]	-	0.11	35%
Pulmonary embolism	3	787	623	1.60 [0.70, 3.64]	-	0.26	0%
Duration of ventilatory post-operatively	3	641	554	-	-1.55 [-3.31, 0.21]	0.08	88%
Multiple organ failure	4	806	807	0.86 [0.35, 2.09]	-	0.74	20%
Deep vein thrombosis	5	820	668	0.84 [0.45, 1.57]	-	0.58	18%
Sepsis	3	641	554	0.69 [0.23, 2.13]	-	0.52	53%
Surgical site infection	3	623	499	0.41 [0.08, 2.19]	-	0.30	41%

EDF: early definitive fixation, LDF: late definitive fixation, RR: risk ratio, WMD: weighted mean difference

The bold value in Table 3 signifies statistically significant lower risk ratio for acute respiratory distress syndrome in the early definitive fixation cohort (EDF)

#### Mortality rates

**Fig. 2:**
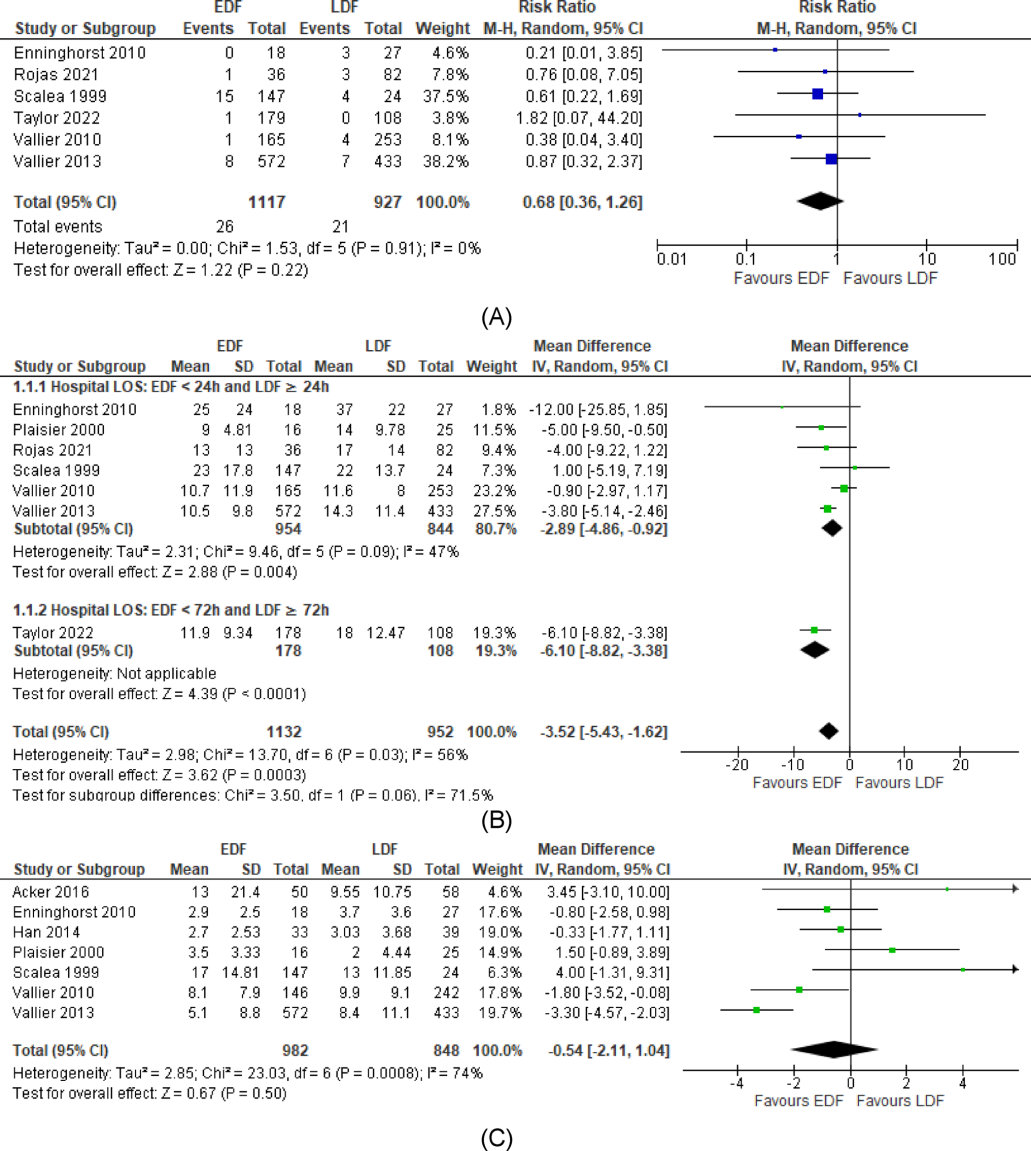
**A** Meta-analysis of association between fracture fixation timing and mortality. **B** Meta-analysis of association between fracture fixation timing and length of hospital stay (days) with subgroup analysis. EDF, early definitive fixation; LDF, late definitive fixation; CI, confidence interval; M-H, Mantel- Haenszel; df, degrees of freedom. **C** Meta-analysis of association between fracture fixation timing and length of ICU stay (days). EDF, early definitive fixation; LDF, late definitive fixation; CI, confidence interval; M-H, Mantel- Haenszel; df, degrees of freedom

Six studies [26, 28–30, 34, 35] reported on their in-hospital mortality between the EDF and LDF groups. No statistically significant association was found between fixation timing and mortality rate as shown in Fig. 2A. (RR = 0.68; 95% CI: 0.36 to 1.26, *p* = 0.22, with low heterogeneity, I² = 0%, *p* = 0.91).

#### Length of hospital stay

Seven studies [26–30, 34, 35] reported length of hospital stay. The pooled results showed a reduction in length of hospital stay in the EDF group of 3.52 days compared to the LDF group (WMD = -3.52; 95% CI: -5.43 to -1.62; *p* < 0.0003 with moderate heterogeneity, I² =56%, *p* = 0.03). This association remained significant after a subgroup analysis to account for differences in time points definition across studies. This is depicted in Fig. 2B.

#### Length of ICU stay

Seven studies [26–31, 33] with similar ISS reported their length of ICU stay. No significant association was found between timing of definitive fracture fixation and length of ICU stay as shown in Fig. 2C (WMD = -0.54; 95% CI: -2.11 to 1.04; *p* = 0.50, with high heterogeneity, I² = 76%, *P* = 0.0008).

#### Pulmonary complications

**Fig. 3:**
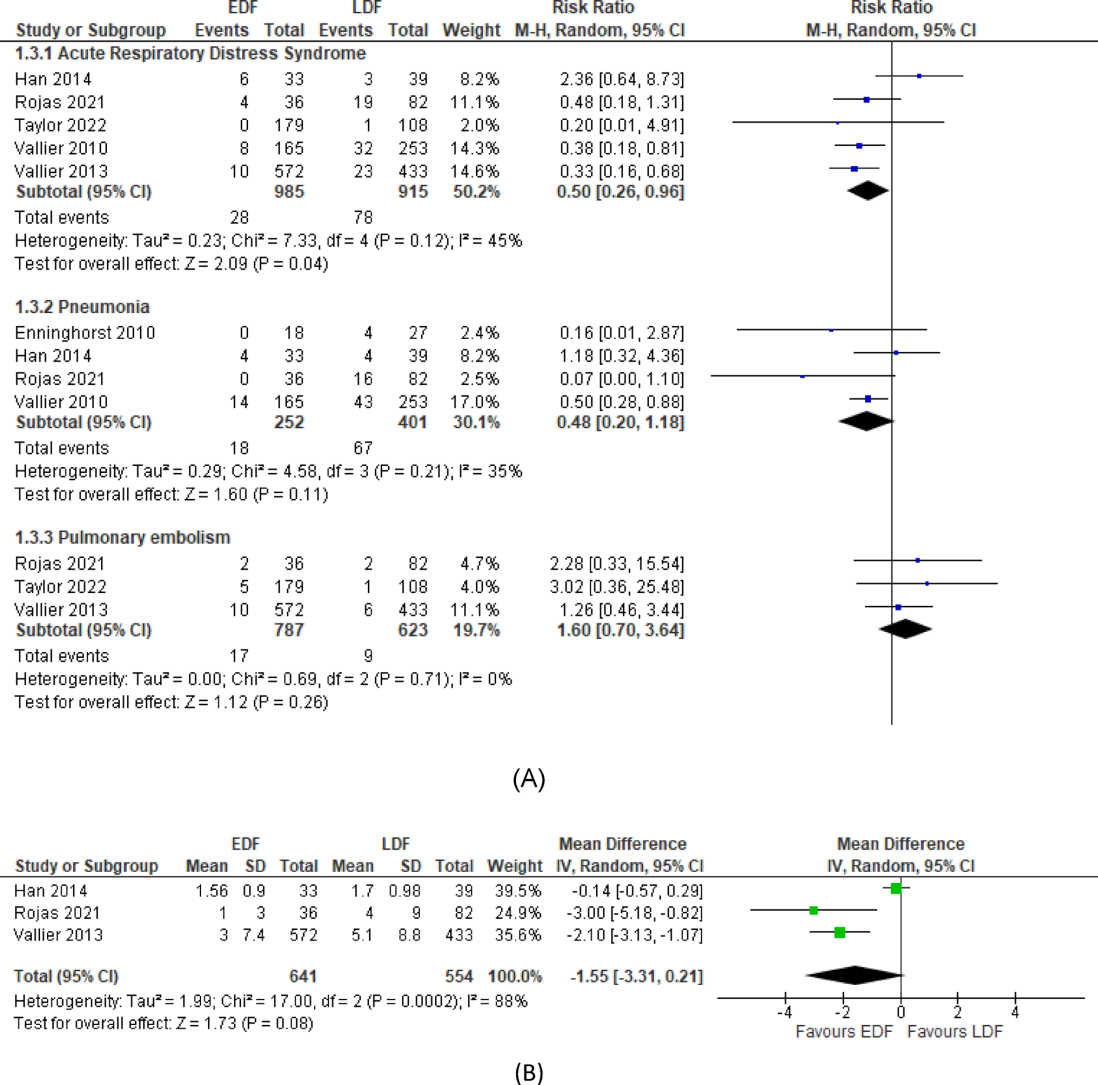
**A** Meta-analysis of association between fracture fixation timing and respiratory complications; (1.3.1) Acute Respiratory Distress Syndrome, (1.3.2) Pneumonia, (1.3.3) Pulmonary embolism. EDF. early definitive fixation; LDF, late definitive fixation; CI, confidence interval; M-H, Mantel- Haenszel; df, degrees of freedom. **B** Duration of ventilatory support (days). EDF. early definitive fixation; LDF, late definitive fixation; CI, confidence interval; M-H, Mantel- Haenszel; df, degrees of freedom

Five studies [28, 30, 31, 34, 35] reported incidence of ARDS and pooled results showed that EDF was associated with a lower rate of ARDS (RR = 0.50; 95% CI: 0.26 to 0.96, *p* = 0.04, with moderate heterogeneity, I² = 45%, *p* = 0.12). Two of those studies adjusted for covariables and injury status and their association of lower ARDS incidence in the EDF group were still significant. Figure 3A depicts these results.

Four studies [28, 29, 31, 34] reported the incidence of pneumonia and pooled results found no significant association with timing of fixation as shown in Fig. 3A (RR = 0.48; 95% CI: 0.20 to 1.18, *p* = 0.11, without significant heterogeneity, I² = 35%, *p* = 0.21).

No significant association was found between timing of definitive fixation and occurrence of pulmonary embolism (PE) (RR = 1.60; 95% CI: 0.70 to 3.64, *p* = 0.26, with low heterogeneity, I² = 0%, *p* = 0.71) across pooled results from three studies [30, 34, 35]. (Fig. 3A).

Three studies [30, 31, 34] reported on duration of ventilatory support post-operatively. No significant association was found across the pooled results. (WMD = -1.55; 95% CI: -3.31 to 0.21, *p* = 0.08, with high heterogeneity, I² = 88%, *p* = 0.0002) (Fig. 3B).

#### Multi-organ failure (MOF)

**Fig. 4:**
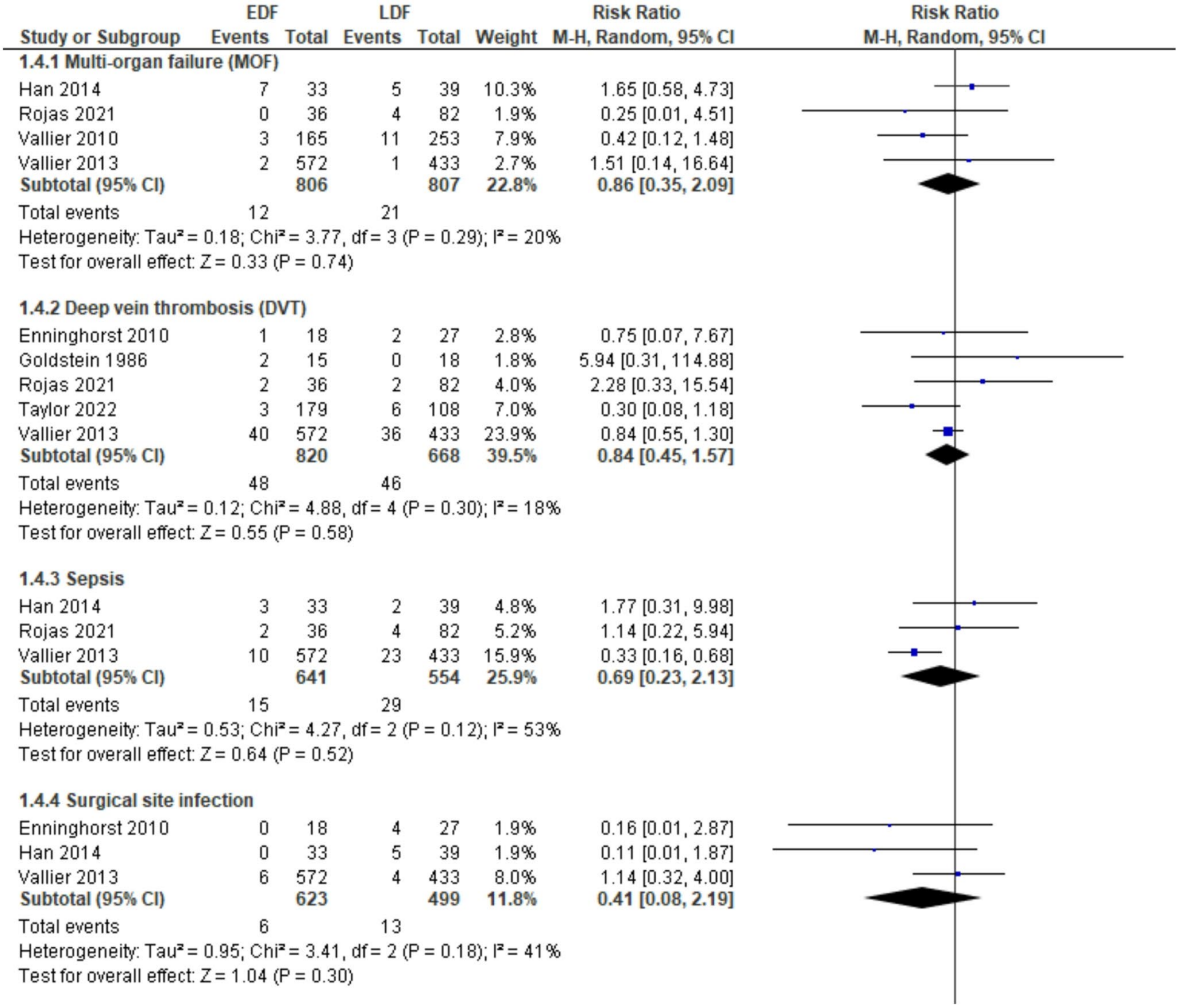
Meta-analysis of association between fracture fixation timing and (1.4.1) Multi-organ failure (MOF), (1.4.2) DVT (deep vein thrombosis), (1.4.3) Sepsis, (1.4.4) Surgical site infection. EDF, early definitive fixation; LDF, late definitive fixation; CI, confidence interval; M-H, Mantel- Haenszel; df, degrees of freedom

Four studies [28, 30, 31, 34] reported the incidence of MOF and their combined results showed no significant difference between the EDF and LDF cohorts as shown in Fig. 4 (RR = 0.86; 95% CI: 0.35 to 2.09, *p* = 0.74, with low heterogeneity, I² = 20%, *p* = 0.29).

#### Deep vein thrombosis (DVT)

Five studies [25, 29, 30, 34, 35] reported on the rates of DVT and found no significant association between the EDF and LDF cohorts, RR = 0.84; 95% CI: 0.45 to 1.57, *p* = 0.58, with low heterogeneity, I² = 18%, *p* = 0.30. The results are shown in Fig. 4.

#### Sepsis

Three studies [30, 31, 34] reported incidence of sepsis and pooled results found no significant difference between EDF and LDF as shown in Fig. 4 (RR = 0.69; 95% CI: 0.23 to 2.13, *p* = 0.52, with moderate heterogeneity, I² = 53%, *p* = 0.12).

#### Surgical site infection

Three studies [29–31] reported the incidence of surgical site infection and pooled results showed no significant difference between EDF and LDF as shown in Fig. 4 (RR = 0.41; 95% CI: 0.08 to 2.19, *p* = 0.30, with moderate heterogeneity, I² = 41%, *p* = 0.18).

## Discussion

To date, the optimal timing for definitive fixation of pelvic ring fractures in major trauma patients continues to generate controversy [9, 13, 36]. For a long time, damage control orthopaedics (DCO) with late definitive fixation has been used in major trauma patients. Several studies have raised concerns of the over utilisation of DCO even in patients who may benefit from early definitive fixation hence avoiding multiple surgeries and longer hospital stays [8, 9, 12, 19].

To our knowledge, this is the first review analysing the effect of timing of definitive fixation in adequately resuscitated polytraumatized patients with pelvic fractures. Our meta-analysis showed no significant difference in postoperative mortality, length of ICU stay, pneumonia, duration of ventilatory support, MOF, DVT, sepsis or surgical site infections between the early and late definitive fixation cohorts. There was however a statistically and clinically significant reduction in length of hospital stay and ARDS in the EDF group. It should be noted that all patients underwent surgery after adequate resuscitation as pointed out by the included studies.

The length of hospital stay was significantly shorter when definitive fixation occurred earlier even after a subgroup analysis to account for variations in the definition of timepoints used across studies. This corroborates with the findings of other studies investigating timing of definitive surgery in major trauma patients with femoral shaft [9, 32, 37] or spine fractures [38–40], suggesting that unnecessary delay in definitive surgery may increase hospital costs [19, 41]. Delay in definitive care has also been associated with an increase in pulmonary complications, deep vein thrombosis, sepsis, skin breakdown [7, 17, 25, 28, 30].

Sharpe et al. [42] studied long-term outcomes after severe pelvic fractures and identified time to definitive pelvic fixation as a predictor of significant impairment in mobility, regardless of age or associated injuries. Prolonged time to definitive fixation led to worse functional outcomes in their patient cohort. The authors suggested that early fixation of the pelvic ring may be a potentially modifiable risk factor for decreased functional outcome in physiologically stable patients. Several other authors have also found that early definitive pelvic fixation is associated with a higher percentage of excellent anatomical reductions and better functional outcomes [17, 18, 27, 43].

### Chest trauma

Vallier et al. performed a subgroup analysis looking at patients with severe chest injury (AIS ≥ 3) and pelvic fractures, no statistical difference was found in pneumonia (*p* = 0.42), ARDS (*p* = 0.21) or MOF (*p* = 0.63) between the EDF (*n* = 46) and LDF (*n* = 78) groups [28]. When patients with severe chest injury were compared with patients without severe chest injury (AIS < 3), the occurrence of complications was higher in the severe chest injury group (*p* < 0.0001). Rojas et al. reached the same conclusion after a multivariate analysis suggesting that chest injury severity plays a larger role in development of pulmonary complications rather than timing of surgery [34]. Bohme et al. investigated patients with severe pelvic fracture (AIS ≥ 3) and severe chest injury (AIS ≥ 4) [44]. Their study failed to demonstrate a correlation between timing of definitive stabilisation and mortality. Furthermore, earlier timing of surgery had no negative effect on perioperative lung function.

Treatment for patients with severe chest trauma should be individualised using a multidisciplinary approach and thorough risk assessment as they are at higher risk of pulmonary complications like ARDS. This should include (a) assessment of the severity of pulmonary dysfunction (presence of lung contusion on chest radiograph or Computed Tomography scan, PaO2/FiO2, positive end-expiratory pressure requirement), (b) haemodynamic status, (c) estimated surgical time and blood loss. A dynamic approach with continuous reassessment with repeat arterial blood gas is advised as pulmonary function can change rapidly after injury [45]. Several studies have advised the use of more precise scoring systems like thoracic trauma score (TTS) over the abbreviated injury score (AIS) as it serves as a better predictor of pulmonary failure [46–49].

### Head injury

There is a paucity of literature investigating patients with head injuries and pelvic fractures [26, 50]. All previous studies include a heterogeneous spectrum of fractures including femoral, pelvic and tibial fractures with conflicting results [51–53]. A previous meta-analysis by Lu et al. investigating timing of extremity fracture fixation in patients with traumatic brain injury (TBI) showed no significant associations between timing of surgery and mortality, need for inotropic support, need and duration of ventilatory support, duration of hospital or ICU stay, changes in GCS scores and neurological complications [54]. On the other hand, Zhang et al. in a meta-analysis on timing of fixation in patients with severe orthopaedic injuries and head injuries noted an increase in mortality, blood loss requiring intraoperative transfusion and hypotension in patients treated early [55].

Hence a definitive conclusion on the management of polytrauma with pelvic fractures and concomitant head injuries cannot be made based on the available evidence. Timing and mode of fracture fixation as well as length of surgery play a critical role in development of complications like intraoperative hypotension, hypoxemia that can be detrimental to cerebral perfusion and place a secondary insult on an already injured brain [51, 52, 54, 56, 57]. Treatment should be based on the patient’s clinical assessment and careful discussion between the neurosurgical and pelvic trauma team rather than timing policies with close monitoring of cerebral pressure perfusion (CPP) to ensure a CPP >70mmHg before, during and after surgery [9, 45, 58].

### Abdominal trauma

Prior to the study by Glass et al. in 2015 [59], timing of definitive fracture fixation was reluctantly delayed in presence of an open abdomen due to a perceived increased risk of infections potentially arising from cross contamination of an exposed cavity. The authors of the study demonstrated the safety and feasibility of definitive fracture fixation in the presence of an open abdomen after a damage control laparotomy and resuscitation in polytrauma patients. 51 patients had pelvic and acetabular fractures out of 81 patients. The study compared two groups: Group 1 had patients receiving definitive fixation with a concurrent open abdomen (mean time to surgery = 4.4 days) and Group 2 had patients receiving definitive fracture fixation after abdominal wall closure (11.8 days; *p* = 0.01). Impressively, they found a statistically significant reduction in orthopaedic surgical site infection requiring revision surgery in Group 1 (3.1%) compared to Group 2 (30.6%; *p* = 0.002). Other outcome measures were similar in both groups. The authors therefore concluded that the timing of definitive fixation should be guided by the physiological response to resuscitation, in analogy to the early appropriate care rather than by timing of abdominal wall closure.

### Liver trauma

Grotz et al. analysed patients with combined pelvic and hepatic injuries with a median ISS of 41. They described this combination of injuries as the “deadly duo” with an overall mortality of 40.7%. 68.4% of deaths occurred within 24 h [60]. Uncontrollable bleeding (52.6%) and severe head trauma (22.7%) accounted for most deaths. Patients having Tile C pelvic fractures and severe liver trauma OIS ≥ 4 (Organ Injury Score) had a mortality rate of 100%. They noted that the severity of liver injury correlated with increasing mortality. Their median time from injury to definitive pelvic surgery was 5 days (2–21 days). The authors concluded that simultaneous assessment and treatment with a rapid and safe decision making is required. They advised the use of damage control techniques to reduce mortality for this “deadly duo” [60].

Overall, the findings from this review suggest that early definitive fixation may be a safe and viable option with no increased risk of complications and mortality. We however believe that the adequacy of resuscitation and the estimate of physiologic reserve should be balanced with the risks of operative fixation in all patients. Various grading systems and concepts of care have been described to help guide the management of polytrauma patients that may benefit from an early definitive versus DCO with late definitive surgery of major fractures [12].

The Early Appropriate Care (EAC) protocol by Vallier et al. recommends definitive fixation of fractures within 36 h of injury provided patients have demonstrated an adequate response to resuscitation [20]. The following parameters were used to define response to resuscitation: improvement in acidosis with lactate < 4.0 mmol/L, pH ≥ 7.25, or base excess ≥ -5.5 mmol/L. Patients were also required to respond to resuscitation without the need for pressor support. It was recently tested prospectively on 335 consecutive patients with ISS ≥ 16; 71 of whom had pelvic ring fractures [32]. Their findings revealed fewer complications (sepsis), shorter hospital and ICU stay in patients treated early with the protocol compared to patients treated over 36 h. The authors also compared their EAC cohort to a historical cohort and controlled for age, timing of fixation and severity of other injuries. Their results showed an improvement in outcomes with the use of the EAC protocol to assess adequacy of resuscitation before surgery. Some limitations of their protocol were the rigid threshold parameters and failure to account for anticipated surgical time and bleeding during the operation [32]. In an analysis of 213 patients with pelvic ring fractures, Probst et al. demonstrated that duration of surgery is an important factor to take into consideration. Initial surgeries lasting more than 3 h were more likely to be associated with higher rates of organ failure in their study [5].

Pape et al. suggested the Safe Definitive Surgery (SDS) concept which is a synthesis of the DCO (LDF) and the Early Total Care concept (EDF), whereby advantages of both strategies can be combined to allow a safe surgery [61]. This concept avoids the dichotomization of patients into either EDF or LDF but rather considers the changes in a patient’s clinical course by re-evaluating and reassessing their physiology to allow for a dynamic classification and adaptation of the treatment strategy. Prospective analyses of this patient centred approach with defined parameters would be a valuable addition to trauma care.

The definition of timing for EDF is heterogeneous in the literature. The most common one used among the included studies were within 24 h of admission. In a previous predictive study, fewer complications were noted when patients were treated within 24 h and to a lesser extent within 48 h of resuscitation [20]. In the UK, the BOAST (British Orthopaedics Association Standards for Trauma) guidelines suggest fixation of all pelvic fractures within 72 h of stabilisation [62]. It also emphasises the importance of trauma network coordination. Patients who require surgical stabilisation but are admitted initially to non-specialist Trauma Units should be referred and safely transferred to a specialist pelvic trauma centre within 24 h. Beyond patient clinical status, other system-level and technical factors also influence timing, such as availability of trained pelvic trauma surgeons, access to interventional radiology, operating theatre capacity, and logistical issues with inter-hospital transfers. These practical realities often dictate whether the BOAST-recommended window of 72 h can be achieved consistently across trauma networks [62].

### Limitations

This systematic review has some limitations. First, the retrospective design of the included studies prevents potential confounding variables to be coherently accounted for. Second, not all studies reported a defined protocol for laboratory measurements to determine adequacy of resuscitation before surgery. Hence the geographical variations in pre-operative resuscitation pathways may have introduced heterogeneity. Furthermore, the reasons for performing definitive fixation at one time point rather than another were not reported in all studies, introducing the possibility of selection bias based on surgeon’s preference, convenience and “risk taking personality”.

Most included cohorts did not explicitly apply a contemporary consensus definition of polytrauma. Modern criteria—often referred to as the *Berlin definition*—require injuries of AIS ≥ 3 in at least two body regions together with ≥ 1 physiologic derangement (hypotension, low GCS, acidosis, coagulopathy, or advanced age) [63, 64]. Adopting a uniform, validated definition in future pelvic-trauma research would improve comparability and external validity. In addition, the proportion of polytrauma patients who sustain pelvic ring injuries—and the distribution of injury severity and required interventions—remains imperfectly characterised across registries; published estimates vary by definitions and case-mix and suggest approximately 10–25% prevalence of pelvic fractures among polytrauma populations [65, 66]. Future prospective, registry-linked studies should report the prevalence, severity mix, and intervention profiles of pelvic injuries within consensus-defined polytrauma cohorts.

## Conclusion

Overall, early definitive fixation of pelvic fractures is a safe and viable option in physiologically stable patients. Caution should however be taken in surgical procedures > 3 h as there is evidence of increased risk of organ failure. Further prospective validation studies are needed to help stratify patients for definitive surgery.

## Supplementary Information

Below is the link to the electronic supplementary material.

Supplementary Material 1

## Data Availability

No datasets were generated or analysed during the current study.
